# Occult endocrine disorders newly diagnosed in patients with post-COVID-19 symptoms

**DOI:** 10.1038/s41598-024-55526-3

**Published:** 2024-03-05

**Authors:** Yasuhiro Nakano, Naruhiko Sunada, Kazuki Tokumasu, Hiroyuki Honda, Yuki Otsuka, Yasue Sakurada, Yui Matsuda, Toru Hasegawa, Daisuke Omura, Kanako Ochi, Miho Yasuda, Hideharu Hagiya, Keigo Ueda, Fumio Otsuka

**Affiliations:** Department of General Medicine, Okayama University Graduate School of Medicine, Dentistry and Pharmaceutical Sciences, 2-5-1 Shikata-cho, Kitaku, Okayama, 700-8558 Japan

**Keywords:** Diabetes mellitus, Endocrine disorders, Long COVID, Metabolic disorders, Thyroid disease, Endocrine system and metabolic diseases, Infectious diseases, Endocrinology

## Abstract

Determination of long COVID requires ruling out alternative diagnoses, but there has been no report on the features of alternative diagnoses. This study was a single-center retrospective study of outpatients who visited our clinic between February 2021 and June 2023 that was carried out to determine the characteristics of alternative diagnoses in patients with post-COVID-19 symptoms. In a total of 731 patients, 50 patients (6.8%) were newly diagnosed with 52 diseases requiring medical intervention, and 16 (32%) of those 50 patients (2.2% of the total) were considered to have priority for treatment of the newly diagnosed disorders over long COVID treatment. The proportion of patients with a new diagnosis increased with advance of age, with 15.7% of the patients aged 60 years or older having a new diagnosis. Endocrine and metabolic diseases and hematological and respiratory diseases were the most common, being detected in eight patients (16%) each. Although 35 of the 52 diseases (67%) were related to their symptoms, endocrine and metabolic diseases were the least associated with specific symptoms. Other disorders that require attention were found especially in elderly patients with symptomatic long COVID. Thus, appropriate assessment and differentiation from alternative diagnoses are necessary for managing long COVID.

## Introduction

SARS-CoV-2, which first appeared in late 2019, had infected more than 769 million people worldwide and more than 33 million people in Japan by July 2023, causing a global pandemic, according to the WHO COVID-19 Dashboard. SARS-CoV-2 not only causes mild to severe respiratory infections in the acute phase (COVID-19) but can also cause prolonged symptoms after recovery, which are called long COVID and also known as post-acute sequelae of COVID-19 or post COVID-19 condition^1^. The reported prevalence of long COVID varies among studies. A study in a large cohort in the Netherlands showed that the effects of COVID-19 accounted for 13% of some symptoms 3–5 months after COVID-19 infection^2^. Long COVID not only causes a deterioration in health conditions but also causes a decline in the quality of life, reduction in the labor force, reduced income and increased medical expenses, and long COVID is thus an important social problem^3^.

Long COVID is defined as prolonged symptoms following SARS-CoV-2 infection that cannot be explained by alternative diagnoses^1^. Long COVID is known to have a variety of symptoms. The most common symptom is fatigue and other symptoms include dyspnea on exertion, palpitations, headaches, sleep disturbances, brain fog, taste and smell disturbances, hair loss and numbness^4,5^. Some patients with these symptoms have conditions that have been noted to be linked to COVID-19 such as postural orthostatic tachycardia syndrome (POTS) and myalgic encephalomyelitis/chronic fatigue syndrome (ME/CFS)^6,7^. We previously reported that a large proportion of patients with long COVID met the criteria for ME/CFS^8^. SARS-CoV-2 enters cells through the angiotensin-converting enzyme 2 (ACE2) receptor, which is expressed in many organs of the body, and can thus cause damage to various organs^9^.

In the endocrine and metabolic systems, COVID-19 has been reported to affect the hypothalamus–pituitary–adrenal gland, thyroid, gonadal function and glucose and lipid metabolism^10^. A recent report has further demonstrated that decreased serum cortisol levels despite strong immune responses is a characteristic of long COVID^11^. We previously reported endocrinological changes in long COVID patients^12–15^. However, not all of the symptoms that occur after COVID-19 are necessarily caused by infection with SARS-CoV-2. It is recommended that patients with post-COVID-19 symptoms undergo appropriate medical evaluations to differentiate their symptoms from symptoms of other diseases^16^.

It is important to consider alternative diagnosis in patients with post-COVID-19 symptoms, and specific procedures for differentiating diseases are provided in some guidelines, but there has been no study on how alternative diagnoses can be established in practice^16^. The aim of this study was to determine the proportion and characteristics of patients with post-COVID-19 symptoms who were diagnosed with diseases other than long COVID, including diseases related to the symptoms and diseases found incidentally, to confirm the importance of appropriate investigation for the management of long COVID.

## Results

**Figure 1:**
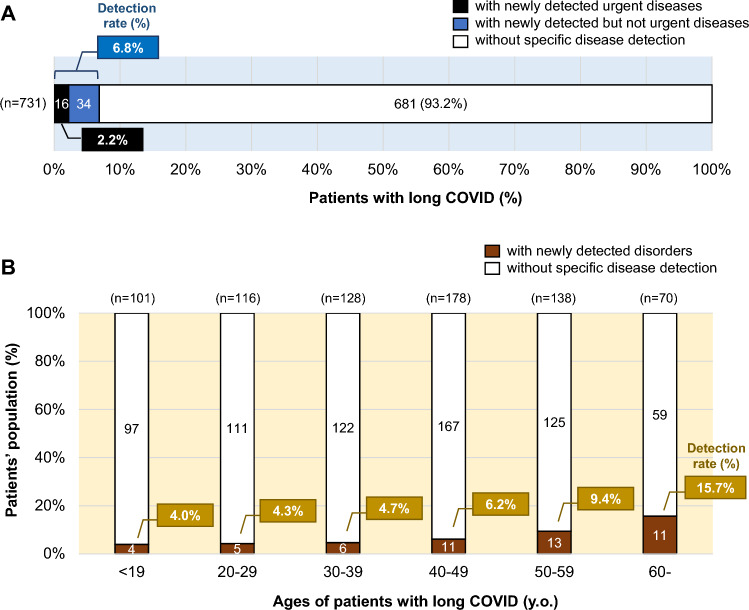
Proportion of long COVID patients with newly detected disorders requiring treatment and/or follow-up. (**A**) Among the 731 long COVID patients, the proportions of patients without newly detected disorders and patients with newly detected disorders including urgent diseases requiring treatment and/or follow-up are shown. (**B**) The age-dependent distribution (%) of long COVID patients with newly detected diseases requiring treatment and/or follow-up is shown.

Data for all of the 734 patients who visited our COVID-19 aftercare outpatient clinic (CAC) during the study period were obtained from medical records. Three patients under the age of 10 years were excluded. Of the 731 patients included in this study, 50 patients (6.8%) were newly diagnosed with 52 diseases requiring treatment and/or follow-up, and 16 (32%) of those 50 patients (2.2% of the total patients) were considered to have priority for treatment of the newly diagnosed condition over long COVID treatment (Fig. 1A).

The clinical backgrounds of the patients with post-COVID-19 symptoms with and those without newly diagnosed diseases (Disease group and Disease-free group) were compared (see Supplementary Table S1 online for all data). The Disease group included 26 females (52%) and 24 males (48%), and the proportions of females and males in the Disease group were similar to those in the Disease-free group. Most of the parameters were not significantly different between the two groups, but the age distributions were significantly different in the two groups (*p* < 0.01). The median ages of patients were 49 years (IQR 37–57.75 years) in the Disease group and 40 years (IQR 25–51 years) in the Disease-free group. The proportions of patients in the Disease group increased with advance of age, the proportion being 15.7% for patients aged 60 years or older (Fig. 1B).

**Figure 2:**
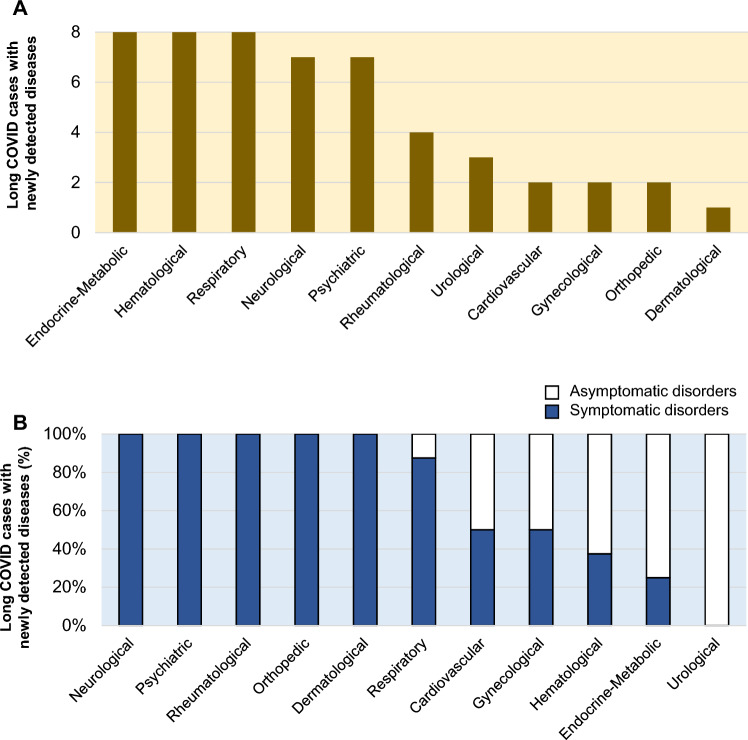
Population of long COVID patients with newly diagnosed diseases based on the organ system. (**A**) The number of long COVID patients with newly diagnosed diseases among the 731 patients is shown in an organ-dependent manner. (**B**) The proportion (%) of patients with newly found diseases related to long COVID symptoms is shown by means of organ-dependent classification.

All newly detected diseases and related symptoms in the long COVID patients are listed in Table 1. As shown in Fig. 2, by organ systems, endocrine and metabolic diseases and hematological and respiratory diseases were the most common, being detected in 8 patients each (8 patients with endocrine and metabolic diseases including 3 patients with type 2 diabetes mellitus, 2 patients with familial hypercholesterolemia, 2 patients with Graves' disease (GD) and 1 patient with reactive hypoglycemia), followed by neurological and psychiatric diseases (7 patients each) (Fig. 2A). Of the 52 diseases, 35 diseases (67%) were associated with symptoms. Respiratory, neurological, and psychiatric disorders were often found in association with symptoms, whereas endocrine and metabolic disorders and hematological and urological disorders were less frequently associated with symptoms (Fig. 2B).

**Table 1:** List of newly diagnosed diseases and related symptoms in each patient.

Organ systems	Diseases	Related symptoms in each patient
Endocrine and metabolic	Familial hypercholesterolemia	–
	Graves’ disease	Dyspnea, Fatigue
	Reactive hypoglycemia	–
	Type 2 diabetes mellitus	–
Hematological	Iron deficiency anemia	Fatigue, Dizziness
	Malignant lymphoma	–
	Monoclonal gammopathy of undetermined significance	–
	Multiple myeloma	–
	Renal anemia	Fatigue
Respiratory	Bronchial asthma	Dyspnea
	Chronic sinusitis	Facial pain, Headache, Nasal congestion
	Exercise-induced asthma	Cough
	Lung cancer	–
	Oral candidasis	Dysgeusia
	Recurrent nerve palsy	Dyspnea
	Subglottic stenosis/granuloma	Dyspnea
Neurological	Cerebrospinal fluid hypovolemia	Dizziness, Headache, Insomnia
	Facial spasm	Facial spasm
	Idiopathic generalized epilepsy	Convulsion
	Migraine	Headache
	Parkinson's disease	Tremor
	Trigeminal neuralgia	Facial pain
Psychiatric	Adjustment disorders	Depressed mood
	Autism spectrum disorder	Insomnia
	Depression	Anxiety, Depressed mood, Fatigue, Insomnia
	Mood dysregulation	Paresthesia
	Panic disorder	Hyperventilation
	Somatic symptoms	Paresthesia
Rheumatological	Dermatomyositis	Cough
	Polymyalgia rheumatica	Myalgia
	Psoriatic arthritis	Arthralgia
Urological	Bladder cancer	–
	Renal cancer	–
Cardiovascular	Microvascular angina pectoris	Chest pain
	Subclavian artery stenosis	–
Gynecological	Adenomyosis of uterus	–
	Endometrial polyp	–
	Premenstrual dysphoric disorder	Irritation
Orthopedic	Compression fracture	Abdominal pain
	Sacral fracture	Lower back pain
Dermatological	Cholinergic urticaria	Pruritus

## Discussion

The present study showed that many patients with post-COVID-19 symptoms, especially elderly patients, have diseases other than COVID-19 that need to be addressed. It was also found that a relatively large proportion of patients with post-COVID-19 symptoms have endocrine and metabolic diseases, which are not associated with post-COVID-19 symptoms in many cases.

It is common that various diseases are detected as people age, and this study showed that this also applies to patients with post-COVID-19 symptoms. COVID-19 has spread worldwide and about one in four people in Japan have been infected with the virus. The reported prevalence of long COVID varies in studies depending on the definition of long COVID. However, considering the large number of COVID-19 cases, it can be assumed that there have been many patients with long COVID. It is likely that all healthcare workers will encounter patients with post-COVID-19 symptoms. Therefore, the results of this study showing that alternative diagnoses are more probable in elderly people indicate the importance for healthcare workers to recognize the possibility of other diseases in elderly patients.

Many endocrine and metabolic disorders were detected in this study, although they had little correlation with symptoms. Malaise, the most common symptom of long COVID, is a symptom that should be considered as a differential diagnosis for endocrine and metabolic disorders such as glucose abnormalities, thyroid dysfunction and adrenal dysfunction. Endocrine and metabolic disorders associated with COVID-19 include type 2 diabetes and thyroid disorders, particularly subacute thyroiditis and euthyroid sick syndrome^17^.

As for metabolic disorders, diabetes mellitus is a risk factor for severe COVID-19, which is associated with new onset of diabetes after COVID-19. A recent cohort study with a median follow-up period of approximately 6 months showed that the incidence of diabetes attributable to SARS-CoV-2 infection was 3–5% and that male sex and severe condition were risk factors for diabetes^18^. As an underlying pathophysiological mechanism, it has been suggested that SARS-CoV-2 can directly infect pancreatic β cells expressing ACE2 receptors, inducing apoptosis and affecting insulin secretion^19^. A recent report has shown that the frequency of childhood obesity increased during the COVID-19 pandemic, and it is anticipated that lifestyle-related diseases such as obesity, fatty liver and diabetes will increase in the future^20^. In the present study, two cases of type 2 diabetes and one case of reactive hypoglycemia were found. Reactive hypoglycemia was thought to reflect glucose intolerance. The pre-COVID-19 status was unknown in this study, and it is therefore not clear whether these diseases were related to COVID-19 infection, but detection of these conditions during CAC outpatient visits is important for patient care. In terms of lipid metabolism, COVID-19 has been shown to affect lipid profiles in the acute phase, but its long-term effects are unknown^10^. The familial hypercholesterolemia found in this study was probably diagnosed incidentally, independently of COVID-19, but careful medical care is important for early detection.

Regarding thyroid diseases, subacute thyroiditis has been reported to have a twofold increased risk of onset in the first 6 months after onset of COVID-19, and subacute thyroiditis after COVID-19 appears to have the same characteristics as those of conventional subacute thyroiditis^21,22^. No cases of subacute thyroiditis were found in this study, probably because subacute thyroiditis is a relatively acute condition and is diagnosed and treated by primary care physicians and internists before referral to the outpatient clinic for long COVID at our university hospital. Euthyroid sick syndrome is less likely to be a problem for patients with long COVID since it resolves after COVID-19 recovery^10^. There have been reports of new cases of GD after COVID-19 and an increase in the incidence of GD during the COVID-19 pandemic^23,24^. Possible mechanisms include aberrant T-cell subtype responses, presence of autoantibodies, regulatory cell dysfunction, and excessive inflammation and immunity due to cytokine overproduction^25^. In patients with post-COVID-19 symptoms, complement activation in patients with brain fog and high serum ferritin levels in patients with ME/CFS after COVID-19 have been reported, suggesting that attention should also be paid to autoimmune abnormalities after COVID-19^26,27^. In the present study, two patients were found to have GD, but the association of GD with COVID-19 is not known because the condition before COVID-19 was unknown. However, it should be emphasized that it is important to check endocrine and metabolic disorders by examinations such as examinations of blood glucose and thyroid function in the management of long COVID.

This study has several limitations. First, this study was a single-center, retrospective study conducted in Japan. Since only patients who were referred to our CAC were included in this study, it is likely that patients with relatively severe symptoms that had persisted for a long time were included. However, it is noteworthy that disease was more frequently found in association with older age in this study regardless of the duration of symptoms or the severity of the acute phase. Secondly, the small number of patients with newly detected disease for each disease makes it impossible to conduct statistical analysis, and only trends can be shown. However, there has been no coherent report of cases of other newly detected diseases in patients with persistent symptoms after COVID-19, and our study provides useful information for clinical practice. Thirdly, the pre-COVID-19 status of the patients was unknown. It is unclear when the disease that was newly discovered in this study developed, and its association with COVID-19 is unclear. In addition, more diseases may be found incidentally as a result of a more thorough systemic examination than that usually performed, for instance, by including the region of gynecological specialty^28^. However, not all symptomatic patients after COVID-19 have long COVID, and the discovery of new or incidental diseases that should be addressed through outpatient visits is beneficial for patients. Fourthly, functional somatic syndrome, which cannot be detected only by clinical examination and the presence of laboratory abnormalities, and aging-related conditions such as postmenopausal syndrome in female patients and late-onset hypogonadism in male patients were not included in the diseases found in this study^12,15^. Therefore, there may be more diseases than those found in this study. Fifthly, the patient's history and complications were not considered. Despite these limitations, this study provides useful information about diseases other than long COVID in patients with post-COVID-19 symptoms.

In conclusion, this study has shown that, among patients with symptoms after COVID-19 infection, the proportion of patients with disorders other than long COVID and the proportion of patients requiring treatment and/or follow-up were unexpectedly high, especially in elderly patients. Endocrine and metabolic disorders were frequently found despite their weak association with long COVID symptoms. Since the diagnosis of long COVID requires the exclusion of alternative diagnoses, appropriate evaluation and careful differential diagnosis of patients with post-COVID-19 symptoms are essential.

## Patients and methods

This study was a retrospective single-center descriptive study. We enrolled and reviewed the medical records of all patients who visited our CAC at the Department of General Medicine, Okayama University Hospital (Japan) from February 14, 2021 to June 30, 2023. This outpatient clinic was established for patients with persistent symptoms after COVID-19. Patients who were under 10 years of age were excluded from the analysis. Well-trained general internists in our CAC carefully examine patients face-to-face and perform necessary laboratory tests and imaging tests as appropriate. From the medical records, we obtained information on age, sex, BMI, severity of COVID-19, history of COVID-19 vaccination, number of days between the onset of COVID-19 and the first visit to the clinic, clinical symptoms of long COVID, and newly diagnosed diseases at the CAC. The severity of COVID-19 during the acute phase was classified according to the criteria defined by the Ministry of Health, Labour and Welfare in Japan^29^.

Among the patients with diseases newly diagnosed at the CAC, patients requiring treatment and/or follow-up for the diseases were included in this study, and patients with biochemical abnormalities alone and diseases such as ME/CFS and POTS that have been shown to be associated with COVID-19 were excluded.

All statistical analyses were conducted by using EZR, version 1.6 (Saitama Medical Center, Jichi Medical University, Saitama, Japan), a graphical user interface for R, version 4.2.2 (The R Foundation for Statistical Computing, Vienna, Austria)^30^. The clinical backgrounds of patients with post-COVID-19 symptoms with and those without newly diagnosed diseases were compared using the Mann–Whitney U test for non-normally distributed continuous variables and Pearson's χ^2^ test or Fisher's exact test for categorical variables. Statistical significance thresholds were defined as **p < 0.01.

This study was approved by the Ethics Committee of Okayama University Hospital (No. 2105-030) and adhered to the tenets of the Declaration of Helsinki. We provided information about the protocol of this study on our website and on the clinic walls of our hospital. The Ethics Committee of Okayama University Hospital waived the need for informed consent from the patients because their data were anonymized, but they could opt out if they preferred.

### Supplementary Information

Supplementary Table S1.

## Data Availability

Detailed data will be available if requested to the corresponding author.

## Abbreviations

ACE2: Angiotensin-converting enzyme 2
BMI: Body mass index
CAC: COVID-19 aftercare clinic
GD: Grave’s disease
ME/CFS: Myalgic encephalomyelitis/chronic fatigue syndrome
POTS: Postural orthostatic tachycardia syndrome

## References

[1] Soriano JB (2022). A clinical case definition of post-COVID-19 condition by a Delphi consensus. Lancet Infect. Dis..

[2] Ballering, A. V., van Zon, S. K. R., Olde Hartman, T. C., Rosmalen, J. G. M. & Lifelines Corona Research, I. Persistence of somatic symptoms after COVID-19 in the Netherlands: an observational cohort study. *Lancet* **400**, 452–461. 10.1016/S0140-6736(22)01214-4 (2022). 10.1016/S0140-6736(22)01214-4 PMC9352274 35934007

[3] Cutler DM (2022). The costs of long COVID. JAMA Health Forum.

[4] Otsuka Y (2021). Clinical characteristics of japanese patients who visited a COVID-19 aftercare clinic for post-acute sequelae of COVID-19/Long COVID. Cureus.

[5] Lopez-Leon S (2021). More than 50 long-term effects of COVID-19: A systematic review and meta-analysis. Sci. Rep..

[6] Mallick D (2023). COVID-19 induced postural orthostatic tachycardia syndrome (POTS): A review. Cureus.

[7] Komaroff AL, Lipkin WI (2023). ME/CFS and long COVID share similar symptoms and biological abnormalities: road map to the literature. Front. Med. (Lausanne).

[8] Tokumasu K (2022). Clinical characteristics of myalgic encephalomyelitis/chronic fatigue syndrome (ME/CFS) diagnosed in patients with long COVID. Medicina (Kaunas).

[9] Oudit GY, Wang K, Viveiros A, Kellner MJ, Penninger JM (2023). Angiotensin-converting enzyme 2-at the heart of the COVID-19 pandemic. Cell.

[10] Bandara T, Deshmukh HA, Abdalla M, Sathyapalan T (2023). Metabolic and endocrine complications of long-COVID-19: A review. Exp. Clin. Endocrinol. Diabetes.

[11] Klein J (2023). Distinguishing features of long COVID identified through immune profiling. Nature.

[12] Sunada N, Otsuka Y, Honda H, Tokumasu K, Otsuka F (2023). Phase-dependent trends of male hypogonadism in long COVID patients. Endocr. J..

[13] Otsuka Y, Otsuka F (2022). Possibility of endocrine dysfunction in post coronavirus disease 2019 (COVID-19) condition. Endocr. J..

[14] Sunada N (2022). Hormonal trends in patients suffering from long COVID symptoms. Endocr. J..

[15] Yamamoto Y (2022). Detection of male hypogonadism in patients with post COVID-19 condition. J. Clin. Med..

[16] Greenhalgh T, Sivan M, Delaney B, Evans R, Milne R (2022). Long covid-an update for primary care. BMJ.

[17] Khan S, Karim M, Gupta V, Goel H, Jain R (2023). A comprehensive review of COVID-19-associated endocrine manifestations. South Med. J..

[18] Naveed Z (2023). Association of COVID-19 infection with incident diabetes. JAMA Netw. Open.

[19] Wu CT (2021). SARS-CoV-2 infects human pancreatic beta cells and elicits beta cell impairment. Cell Metab..

[20] Fujiwara S (2023). Trends in childhood obesity in Japan: A nationwide observational study from 2012 to 2021. Clin. Obes..

[21] Lee J, Seo GH, Song K (2023). Beyond acute COVID-19: Investigating the incidence of subacute thyroiditis in long COVID-19 in Korea. Endocrinol. Metab. (Seoul).

[22] Meftah E (2023). Subacute thyroiditis following COVID-19: A systematic review. Front. Endocrinol. (Lausanne).

[23] Brancatella A, Viola N, Santini F, Latrofa F (2023). COVID-induced thyroid autoimmunity. Best Pract. Res. Clin. Endocrinol. Metab..

[24] Barajas Galindo DE (2023). Increased incidence of graves' disease during the SARS-CoV2 pandemic. Clin. Endocrinol. (Oxf).

[25] Mohammadi B (2023). COVID-19-induced autoimmune thyroiditis: Exploring molecular mechanisms. J. Med. Virol..

[26] Yamamoto Y (2023). Utility of serum ferritin for predicting myalgic encephalomyelitis/chronic fatigue syndrome in patients with long COVID. J. Clin. Med..

[27] Hagiya H (2024). Relevance of complement immunity with brain fog in patients with long COVID. J. Infect. Chemother..

[28] Sakurada Y (2024). Clinical characteristics of female long COVID patients with menstrual symptoms: A retrospective study from a Japanese outpatient clinic. J. Psychosom. Obstet. Gynaecol..

[29] Kato Y (2021). Case management of COVID-19 (secondary version). JMA J..

[30] Kanda Y (2013). Investigation of the freely available easy-to-use software 'EZR' for medical statistics. Bone Marrow Transpl..

