# Supplementary material for: Occult endocrine disorders newly diagnosed in patients with post-COVID-19 symptoms
Source: Sci Rep. 2024 Mar 5;14:5446. doi: 10.1038/s41598-024-55526-3 (PMC10914791; doi:10.1038/s41598-024-55526-3)
Supplement: Supplementary file 1 — Supplementary Table S1. [file 41598_2024_55526_MOESM1_ESM.docx]

**Supplementary Table S1. Backgrounds of the patients with post-COVID-19 symptoms with and without newly diagnosed diseases.**

|  | **Disease-free group** |  | **Disease group** |  | ***p*-Value** |
| --- | --- | --- | --- | --- | --- |
|  | (n=681) |  | (n=50) |  |  |
| Sex |  |  |  |  | 0.8 |
| Female | 374 (55%) |  | 26 (52%) |  |  |
| Male | 307 (45%) |  | 24 (48%) |  |  |
| Age distribution |  |  |  |  |  |
| Median (IQR) | 40 (25-51) |  | 49 (37-57.75) |  | <0.01** |
| <19 | 97 (14%) |  | 4 (8%) |  | 0.306 |
| 20-29 | 111 (16%) |  | 5 (10%) |  | 0.329 |
| 30-39 | 122 (18%) |  | 6 (12%) |  | 0.385 |
| 40-49 | 167 (25%) |  | 11 (22%) |  | 0.818 |
| 50-59 | 125 (18%) |  | 13 (26%) |  | 0.252 |
| 60- | 59 (9%) |  | 11 (22%) |  | <0.01** |
| BMI |  |  |  |  |  |
| Median (IQR) | 22.4 (20.3-25.9) |  | 22.5 (19.6-25.4) |  | 0.855 |
| <25 | 475 (70%) |  | 36 (72%) |  | 0.861 |
| 25-30 | 145 (21%) |  | 7 (14%) |  | 0.296 |
| >=30 | 58 (9%) |  | 7 (14%) |  | 0.195 |
| unknown | 3 (0%) |  | 0 (0%) |  |  |
| Severity of the acute phase of COVID-19 | |  |  |  |  |
| Mild | 603 (89%) |  | 43 (86%) |  | 0.754 |
| Moderate-I | 33 (5%) |  | 1 (2%) |  | 0.723 |
| Moderate-II | 36 (5%) |  | 5 (10%) |  | 0.191 |
| Severe | 8 (1%) |  | 1 (2%) |  | 0.473 |
| unknown | 1 (0%) |  | 0 (0%) |  |  |
| COVID-19 vaccinations |  |  |  |  |  |
| None | 232 (34%) |  | 16 (32%) |  | 0.886 |
| 1 dose | 29 (4%) |  | 1 (2%) |  | 0.714 |
| 2 doses | 212 (31%) |  | 19 (38%) |  | 0.395 |
| >= 3 doses | 199 (29%) |  | 13 (26%) |  | 0.747 |
| unknown | 9 (1%) |  | 1 (2%) |  | 0.51 |
| Duration after the onset of COVID-19 to the first visit | | |  |  |  |
| < 1 month | 4 (1%) |  | 1 (2%) |  | 0.299 |
| 1-2 months | 148 (22%) |  | 12 (24%) |  | 0.844 |
| 2-3 months | 166 (24%) |  | 12 (24%) |  | 1 |
| 3-4 months | 121 (18%) |  | 7 (14%) |  | 0.628 |
| 4-5 months | 83 (12%) |  | 8 (16%) |  | 0.571 |
| 5-6 months | 39 (6%) |  | 3 (6%) |  | 1 |
| >= 6 months | 120 (18%) |  | 7 (14%) |  | 0.646 |

Medians [IQR: interquartile ranges] and percentages (%) are shown. BMI: body mass index. Pearson's χ2 test, Fisher's exact test and the Mann-Whitney U test were performed when appropriate for statistics, and ***p* < 0.01 indicates statistically significant differences.
